# Identification of the Histone Deacetylases Gene Family in Hemp Reveals Genes Regulating Cannabinoids Synthesis

**DOI:** 10.3389/fpls.2021.755494

**Published:** 2021-10-20

**Authors:** Liu Yang, Xiangxiao Meng, Shilin Chen, Jun Li, Wei Sun, Weiqiang Chen, Sifan Wang, Huihua Wan, Guangtao Qian, Xiaozhe Yi, Juncan Li, Yaqin Zheng, Ming Luo, Shanshan Chen, Xia Liu, Yaolei Mi

**Affiliations:** ^1^School of Chemistry, Chemical Engineering and Life Sciences, Wuhan University of Technology, Wuhan, China; ^2^Key Laboratory of Beijing for Identification and Safety Evaluation of Chinese Medicine, Institute of Chinese Materia Medica, China Academy of Chinese Medical Sciences, Beijing, China; ^3^Key Laboratory of Saline-alkali Vegetation Ecology Restoration, Ministry of Education, College of Life Sciences, Northeast Forestry University, Harbin, China; ^4^Guangdong Provincial Key Laboratory of Applied Botany, South China Botanical Garden, Center of Economic Botany, Core Botanical Gardens, Chinese Academy of Sciences, Guangzhou, China

**Keywords:** histone deacetylase, *Cannabis sativa*, gene family, bioinformatics, genome-wide

## Abstract

Histone deacetylases (HDACs) play crucial roles nearly in all aspects of plant biology, including stress responses, development and growth, and regulation of secondary metabolite biosynthesis. The molecular functions of HDACs have been explored in depth in *Arabidopsis thaliana*, while little research has been reported in the medicinal plant *Cannabis sativa* L. Here, we excavated 14 *CsHDAC* genes of *C. sativa* L that were divided into three relatively conserved subfamilies, including RPD3/HDA1 (10 genes), SIR2 (2 genes), and HD2 (2 genes). Genes associated with the biosynthesis of bioactive constituents were identified by combining the distribution of cannabinoids with the expression pattern of HDAC genes in various organs. Using qRT-PCR and transcription group analysis, we verified the expression of candidate genes in different tissues. We found that the histone inhibitor Trichostatin A (TSA) affected the expression of key genes in the cannabinoid metabolism pathway and the accumulation of synthetic precursors, which indirectly indicates that histone inhibitor may regulate the synthesis of active substances in *C. sativa* L.

## Introduction

Chromatin consists of nucleosomes, each of which is composed of an octamer of four key histones—H2A, H2B, H3, and H4, each of which is a dimer—around which is wrapped a strand of DNA (Yang and Nielsen, 2000; Lusser et al., 2001). Histone acetylases (HAT) are enzymes, which catalyzes the acetylation of histones, and histone deacetylase (HDAC) catalyzes the deacetylation of histones (Yang and Seto, 2007; Wang et al., 2009). When histones are acetylated, they release DNA due to the decrease in the affinity between histones and DNA, and subsequently promote the binding of transcription factors to DNA (Kurdistani and Grunstein, 2003). However, these effects are counteracted by histone deacetylases (Liu et al., 2012). HAT and HDAC coexist in various tissues and organs of animals and plants and interact to regulate the availability of DNA (Graessle et al., 2001; Haigis and Guarente, 2006). The classification and function of HDACs in humans and yeast have been studied in depth (Brownell et al., 1996; Song and Galbraith, 2006). In yeast, the HDAC gene family is divided into two subfamilies according to the characteristics of the conserved domain sequences RPD3/HDA1 (reduced potassium dependency 3) and SIR2 (silent information regulator 2; Frye, 2000; Guarente, 2000). Histone deacetylation can directly affect the level of transcription by altering the structure of chromatin (Brownell et al., 1996; Pandey et al., 2002). Both subfamilies have also been studied in plants (Zhang et al., 2020). For example, *AtHDA1*, *AtSNLs,* and *AtHDA19* regulate flowering time by forming complexes (Lee et al., 2016); *AtHDA19* and *AtHSL1* together suppress the expression of seed maturation genes (Zhou et al., 2013). In rice, *OsIDS1* physically interacts with the transcriptional corepressors *OsTPR1* and *OsHDA1* (Cheng et al., 2018). Additionally, another subfamily of HDAC, HD2 (Histone Deacetylase 2), was shown to be plant-specific (Gu et al., 2011; Nicolas-Francès et al., 2018). In Poplar lignin, the *PtHDT1* mutant of *Populus tomentosa*, an HD2 subfamily member, has thinner stem nodes, more wood fiber cells, thicker wood fiber cell walls, and higher lignin than the wild type (Kornet and Scheres, 2009). *PtHDT1* interacts with *PtMYB* to regulate the development of xylem and cambium (Zhang, 2018). In banana, *MaERF1* recruits *MaHDA1* to represses the expression of *MaACO1*, thereby negatively regulating ethylene biosynthesis (Han et al., 2016; Zheng et al., 2020). Therefore, HDAC families exert vital effects on the synthesis of secondary metabolites.

**Figure 1:**
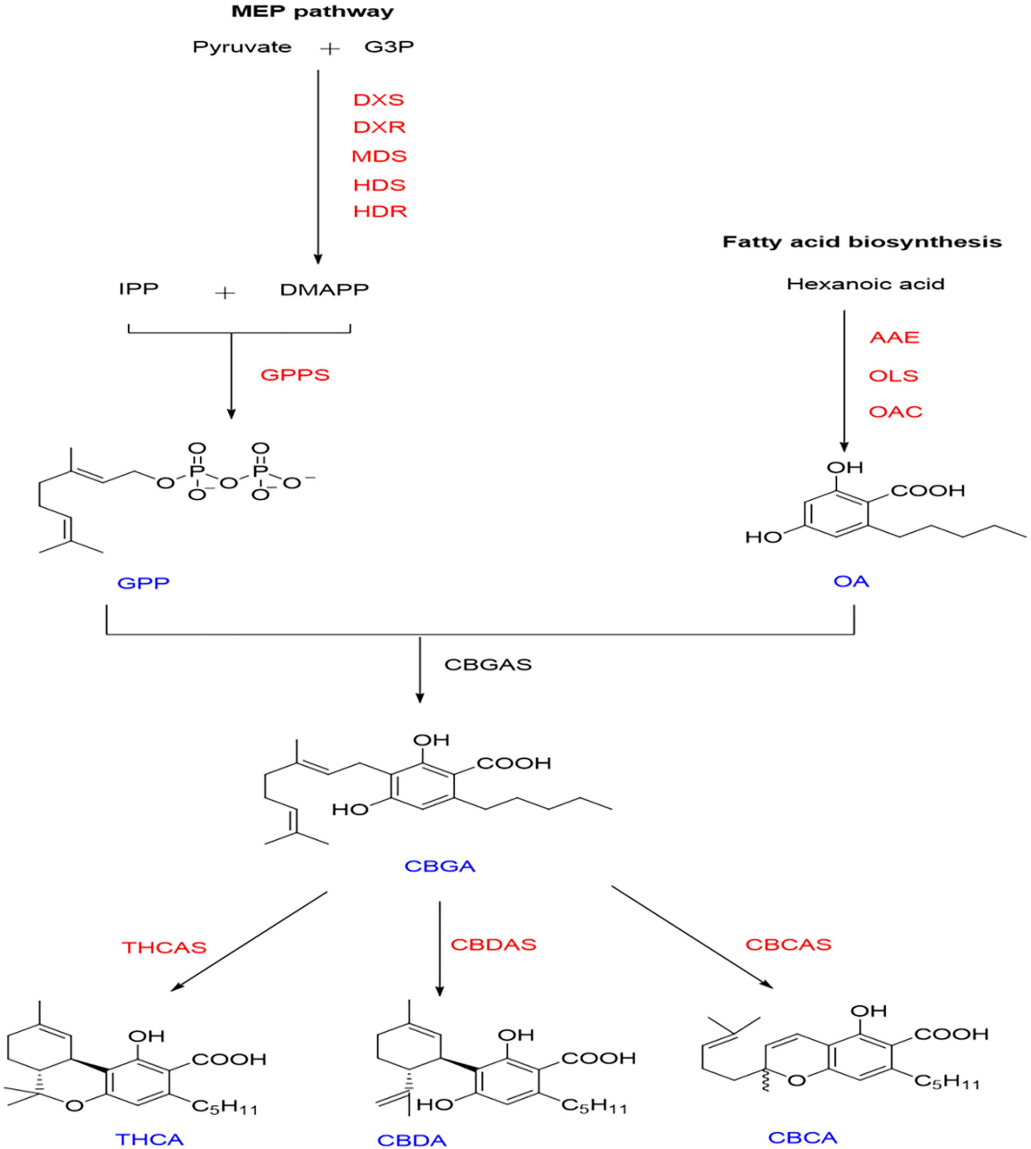
Synthetic pathway of cannabinoids. Intermediates and final products are shown in blue. Enzyme names are shown in red. Fatty acid pathway—AAE, Acyl activating enzyme; OAC, Olivetolic acid cyclase; and OLS, Olivetol synthase. Methylerythritol phosphate (MEP) pathway—DXS, 1-deoxy-d-xylulose 5 phosphate DXP synthase; DXR, DXP reductase; MDS, 2-C-methyl-pyrophosphate synthase; HDS, (E)-4-Hydroxy-3-methyl-but-2-enyIpyrophosphate (HMB-PP) synthase; HDR, HMB-PP reductase; and GPPS: Geranyl diphosphate synthase. Intermediates and final products—GPP, Geranyl diphosphate; OA, Olivetolic acid; CBGA, Cannabigerolic acid; THCA, Δ9-Tetrahydrocannabinolic acid A; CBDA, Cannabidiolic acid; and CBCA, Cannabichromenic acid.

*Cannabis sativa* (Cannabinaceae) is an annual herb, mostly dioecious (Zuardi, 2006). This plant has been used in many areas including food, fiber, cosmetics, and medicine (Schachtsiek et al., 2018). Cannabinoids, the main components found in *C. sativa.* L, include CBD and THC (Marks et al., 2009; Vindenes, 2017). *C. sativa* is divided into hemp and marijuana, according to the content of tetrahydrocannabinol (THC; Morimoto et al., 1997; Vindenes, 2017). CBD has anti-vomiting, analgesic, anti-inflammatory, anti-spasmodic, anti-cancer, and other activities (De Meijer and Hammond, 2005). For instance, epidiolex (purified CBD) is an oral drug approved for treating epilepsy. However, THC is a neuroactive compound and is addictive (Pacher et al., 2006; Burstein, 2015). In China, the cultivation of hemp with a THC content higher than 0.3% is prohibited. Therefore, our lab is committed to cultivating high-quality varieties of *C. sativa* with high CBD and low THC content. This aim will be achieved by studying the molecular mechanism related to the production of CBD to increase natural yield and response to external environmental stress. The biosynthetic pathway of cannabinoids has been elucidated (Lydon et al., 1987; Booth et al., 2020; Figure 1). However, the molecular mechanism of regulation of cannabinoid synthesis has yet to be clearly resolved. Posttranscriptional modification plays a pivotal role in the biosynthetic pathway of active substances in medicinal plants (Appendino et al., 2008; VanBakel et al., 2011; Luo et al., 2019). We aimed to systemically summarize the members of the HDAC family at the genome-scale level and explore histone deacetylation the effects of posttranscriptional modification on cannabinoid synthesis. In this study, we identified 14 HDACs in *C. sativa* genome and studied their structural characteristics, subcellular localization, alternative splicing events, and differential expression in different tissues and organs. We treated *C. sativa* L seedlings with histone inhibitors and measured the expression of key genes and the accumulation of precursor substances (olivetolic acid-OA and geranyl diphosphate-GPP). Based on these studies, we explored the role of deacetylation in the cannabinoid synthesis pathway.

## Materials and Methods

### Plant Materials and Growth Condition

In this study, we used the hemp variety Diku (DK), a female plant crossing Purple Kush with Dinamed Autoflowering CBD. This variety has a short growth cycle and is a variety with high CBD and low THC content. It is an excellent reference variety for cannabis breeding. The plants were cultivated in the experimental field of the Institute of Chinese Materia Medica of the Chinese Academy of Chinese Medical Sciences, China. Flowers, bracts, leaves, stems, seeds, and roots of hemp were collected to analyze the expression patterns of HDAC genes. Hemp seeds were cultured in MS medium for 3weeks. After the seedlings grew, they were transferred to an MS medium containing 0-μm (DMSO matrix), 0.25-μm, or 1.25-μm Trichostatin A (TSA) and cultured for 1week to analyze the effect of TSA on the HDAC gene of hemp. All sample materials collected in this study were frozen in liquid nitrogen immediately and then reserved at −80°C for further analysis.

### Data Sources

The genomic data (GCA_900626175.1) of hemp variety female CRBRX, with a high-CBDA cultivar (15% CBDA and 0.3% THCA) used in this study, were downloaded from the NCBI^1^ database. *Arabidopsis thaliana* and soybean HDAC genetic data were obtained from the PlantTFDB database.^2^ We measured the transcriptome of five different tissues and organs of Diku. The transcriptome data of its flowers, bracts, stems, leaves, and seeds were available at NCBI (NCBI accession number: flowers: SAMN16122886~SAMN16122888; bracts: SAMN16122880~SAMN16122882; stems: SAMN16122883~SAMN16122885; leaves: SAMN16122889~SAMN1612281; and seeds: SAMN20474456~SAMN20474458).

### Basic Information and Characteristics of CsHDACs

We downloaded the full gene set and annotation files of *C. sativa* (GCA_900626175.1), *Glycine max* (GCF_000004515), and *A. thaliana* (GCA_000005425.2) from the NCBI Web site. We extracted the CDS sequence and protein sequence of hemp using the TBtools software (Chen et al., 2020). Using the protein sequence of AtHDAC and GmHDAC as query in BLASTp (score value of ≥100, e-value ≤ e^−10^), we ran the extracted hemp data against the AtHDAC sequence to identify potential HDACs in hemp. Then, we initially established identity-reliable domains by manually searching the NCBI reserved domain database.^3^ Finally, 14 HDAC family members were obtained. The basic characteristics, such as the protein molecular weight, isoelectric point, and amino acid number, were analyzed on the ExPASy Web site.^4^

### Phylogenetic, Conserved Motif Analyses, Gene Structure, Chromosomal Locations, and Syntenic Analyses of CsHDACs

CsHDAC, GmHDAC, and AtHDAC domain amino acid sequences were aligned using MEGA 7.0 software. The neighbor-joining (NJ) adjacency method was used to build a phylogenetic tree against the *A. thaliana* and *G. max*. The bootstrap test was repeated 1,000 times, and the BLASTp cutoff was set as value of *E*<1×10^−10^. The structure of the CsHDACs and their localization on the chromosomes were visualized using TBtools (Chen et al., 2020). The conserved motifs of CsHDAC proteins were carried out using MEME.^5^ The parameters are set as follows: The maximum number of themes is 20, and the best theme width is 10–200. Conserved structural domains were analyzed using NCBI-CDD.^6^ Subcellular localization was predicted using the wolf PSORT Web site.^7^ Sequences 2000bp upstream of the start codon of each CsHDAC gene were extracted, and the distribution of the *cis*-elements in the promoter regions was analyzed using PlantCARE software.^8^ We downloaded the chromosomal locations of 14 HDAC genes in the *C. sativa* genome from the NCBI.^9^ The HDAC gene locations were then represented using TBtools (Chen et al., 2020). Multiple collinear scanning toolkits (MCScanX) were used to detect gene duplication events (Wang et al., 2012).

### Gene Expression in Different Tissues and Alternative Splicing Analysis

Differential expression analysis of CsHDACs and all isoforms of CsHDACs were performed, and heat maps were created using TBtools software. In short, SpliceMap was used to detect the junctions of Illumina short reads obtained from samples, and IDP was then used to predict and detect isoforms by integrating both Illumina short reads and SMRT long reads. The results of IDP show the sites where isoforms are located on the genome. Integrative Genomics Viewer was used to visualize the isoform structure that may exist for each HDAC gene family member.^10^ Specific steps were referred to in our previous research (Gao et al., 2019; Xu et al., 2020). We took the original annotated genes as reference isoforms (ref isoforms). The other isoforms were divided into intron retained (IR), an exon skipping (ES), and alternative 3′ splice site (AA). Isoforms that did not belong to the aforementioned three types were defined as “others.”

### RNA Isolation and qRT-PCR

According to the manufacturer’s instructions, we isolated total RNA from samples (Tiangen Biotech, Beijing, China). Using a Fast Quant RT Kit, the first cDNA strand was synthesized (Tiangen Biotech). The specific primers for qRT-PCR were designed using NCBI-Primer blast (Table 1). CFX96^™^ real-time system (Bio-Rad, Hercules, CA, United States) was used for qRT-PCR. Program: 95°C for 2min, followed by 40cycles of 95°C for 15s, 56°C for 35s, and 72°C for 15s. A melting curve was generated and analyzed. The reference gene in this study was *EF1-α* (Nicot et al., 2005). Each sample was in triplicate experiments.

**Table 1:** qRT-PCR primers for genes.

Protein name	Forward primer	Reverse primer
CsHDA1	TCCCTGGTACCGGAGACATAG	CACTGAAGAACCACAGCCCC
CsHDA2	AGGTTGGAGGAACTATTGTGGC	CACAAAATCCCCCTCCTCTCC
CsHDA3	TGGAAAACCAGCTCTCACTTCT	CCCTCCACAAAACATCTTACCC
CsHDA4	GGGCTTGCCATAGTTGATTCC	GCATAACGAGCAGCTATTGCC
CsHDA5	GCGTCAAAAATGGGTTTGCCC	CAATGAGCACCTTCCTAGCCC
CsHDA6	TGGAGATGGTGTTGAGGAAGC	GGCCGAAAAAGACCACGAAAA
CsHDA7	CCGGTGATGTCAAGGATTCAGG	GAAGCAGCCTAAGCGATCTCC
CsHDA8	CTGCTTCCTTAACAATGCTGGTC	AGGATGAGATGAACCCCATGAAC
CsHDA9	TGCCCAAGGGAAAGTTGTGTT	GCAAGTGTAGGCCAGAATGGA
CsHDA10	GTTGGGTAGAGGCGAAGACC	ACGGCTACACAATACCTCCT
CsHDT1	TGAAAACTTGCAGTCTGGAGAAG	TGAATCGTTTTCTTCATCACCAC
CsHDT2	TCCGATGAGGAAGAAGAAGTCC	GCTTGGCTCCACAACCTTAACT
CsSRT1	ATGTTTGGGGACGAGCCTG	ATCATGGCACCCGAAACTACC
CsSRT2	GGAGTTTATTCGCTCAAGCCG	AGAGCGATATGAGCAGCACC

### Sample Preparation and UPLC-ESI-MS/MS Conditions

After adding liquid nitrogen in a mortar, leaves of hemp seedlings were crushed into a uniform powder. An ultrasonic ice water bath was used to dissolve approximately 0.1-g sample powder in 10.0-ml 95% aqueous methanol for 30min. Approximately 12,000rpm was applied for 10min at 4°C to centrifuge the solution; then, the supernatant was filtered by a 0.22-μm hydrophilic organic nylon microporous membrane.

The Agilent UPLC 1290II–G6400 triple quadrupole mass spectrometer (QQQ; Agilent Technologies, Santa Clara, CA, United States) was used to determine the relative quality of synthetic precursors. We used the peak area to express the relative mass under each condition.

A bidirectional solvent delivery system, an autosampler, and a column compartment were used in the UPLC. A column (2.1 ^*^ 100mm, 1.8μm) of C18 was used for chromatographic separations at a temperature of 40°C. Mobile phase A contained water containing 0.1% formic acid; phase B is 100% methanol. Elution was proceeded linearly at a flow rate of 0.3ml/min. In gradients, these settings were used as: 5% B→70%B (0–2min); 70% B→100% B (2–10min); 100% B (10–13min); 100% B→5% B(13–14min); and 5% B (14–15min). Autosampler was set to 4°C and 3-μl sample volume was injected. The experiment was performed in triplicate. The MRM diagrams of OA, GPP, and their standard products are shown in Supplementary Figure S1.

### Statistical Analysis

All the data were analyzed by the Prism 8 Statistics programs, and the means were compared by the least significant difference test at the 0.05 and 0.01 level of significance.

## Results

### Basic Information and Characteristics of CsHDACs

In our study, 14 HDAC genes were obtained, and RPD3/HDA1 subfamily contained 10 members, named *CsHDA1*–*CsHDA10*; two members of HD2 subfamily named *CsHDT1*–*CsHDT2*; and two members of SIR2 subfamily named *CsSRT1*–*CsSRT2*. Their sequences ranged in length from 432 to 1953 bp. The molecular weight of their translated protein ranged from 21702.17 to 56624.95Da, and they had between 203 and 504 amino acids. The other characteristics, such as theoretical IP and subcellular localization, were shown in Table 2.

**Table 2:** Description of the HDACs in *C. sativa*.

Gene ID	Protein id	Protein name	Protein Length (aa)	Molecular Weight (Da)	Theoretical IP	Subcellular localization
LOC115702813	XP_030486110.1	CsHDA1	494	55699.25	5.00	Cyto. Nuc.
LOC115707169	XP_030490898.1	CsHDA2	388	43016.59	8.07	Nuc
LOC115710981	XP_030495193.1	CsHDA3	203	21702.71	6.02	Nuc
LOC115707945	XP_030491922.1	CsHDA4	422	45486.66	5.95	Nuc
LOC115711299	XP_030495493.1	CsHDA5	494	53597.21	5.48	Nuc
LOC115710180	XP_030494383.1	CsHDA6	422	52277.81	5.47	Cyto. Nuc
LOC115719134	XP_030503922.1	CsHDA7	432	49345.45	5.15	Nuc
LOC115718595	XP_030503266.1	CsHDA8	387	41888.25	5.47	Cyto. Nuc
LOC115709622	XP_030493624.1	CsHDA9	504	56624.95	5.81	Nuc
LOC115703044	XP_030486387.1	CsHDA10	464	52277.81	5.47	Cyto. Nuc
LOC115711643	XP_030495876.1	CsHDT1	296	31610.11	4.59	Nuc
LOC115719367	XP_030504240.1	CsHDT2	311	33030.00	4.63	Nuc
LOC115695686	XP_030478610.1	CsSRT1	484	53608.19	9.04	Chlo. Nuc
LOC115714802	XP_030499415.1	CsSRT2	397	43486.62	8.37	Chlo. Nuc

### Evolutionary Analysis of the CsHDAC Family

**Figure 2:**
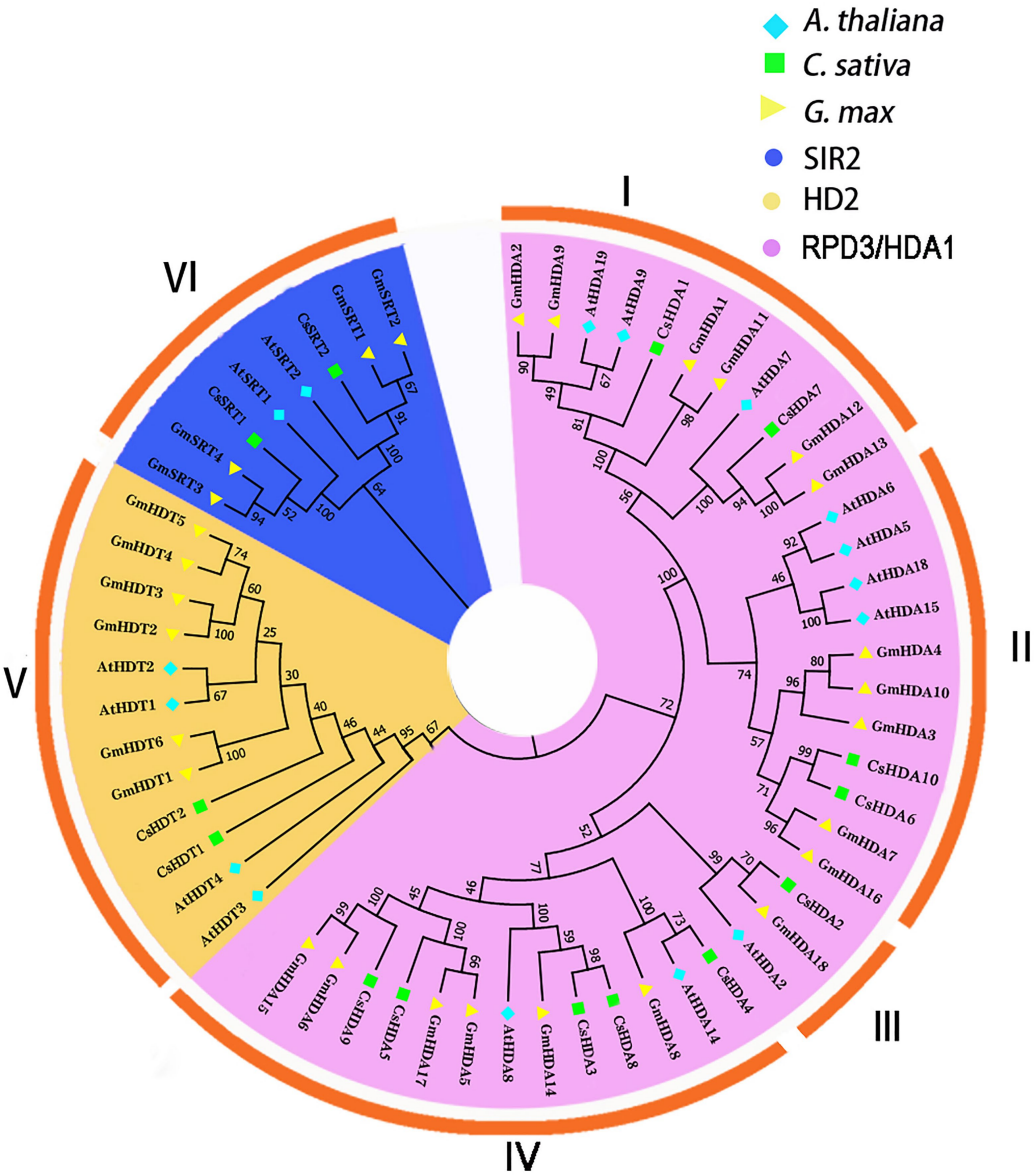
An unrooted phylogenetic tree was constructed with the HDAC domain amino acid sequences of *Arabidopsis thaliana, Cannabis sativa*, and *Glycine max.* A neighbor-joining tree was constructed using MEGA7 with 1,000 bootstrap replicates. The tree was divided into six groups that contained the RPD3/HDA1, SIR2, and HD2 subfamilies (I–IV: RPD3/HDA1; V: HD2; and VI: SIR2). The blue rhombus represents *A. thaliana*; the green square indicates *C. sativa*; and the yellow triangle represents *G. max.* The purple background shows the SIR2 subfamily; the yellow background refers to the HD2 subfamily; and the blue background represents the RPD3/HDA1 subfamily.

To demonstrate the evolutionary relationships of CsHDAC in plants, we constructed a phylogenetic tree using amino acid sequences from model plant Arabidopsis, model food crop soybean, and hemp. The CsHDAC family was categorized into three subfamilies based on the difference in sequence features and the number of HDAC domains, SIR2, HD2, and RPD3/HDA1, with 2, 2, and 10 members, respectively (Zhang et al., 2020). Clades I–IV represented the RPD3/HDA1 subfamily, which is a type of Zn^+^-dependent histone deacetylase. Clade V represented the HD2 subfamily, which are plant-specific HDACs. Clade VI represented the SIR2 subfamily, a type of nicotinamide adenine dinucleotide (NAD^+^)-dependent HDACs. The 10 members of RPD3/HDA1 subfamilies were further divided into four subgroups based on the similarities of the HDAC domain amino acid sequences, with Subgroup I including CsHDA1 and CsHDA7, Subgroup II including CsHDA6 and CsHDA10, Subgroup III including CsHDA2, and Subgroup IV including CsHDA3-5 and CsHDA8-9 (Figure 2).

### Conserved Motifs and Gene Structure of CsHDACs

**Figure 3:**
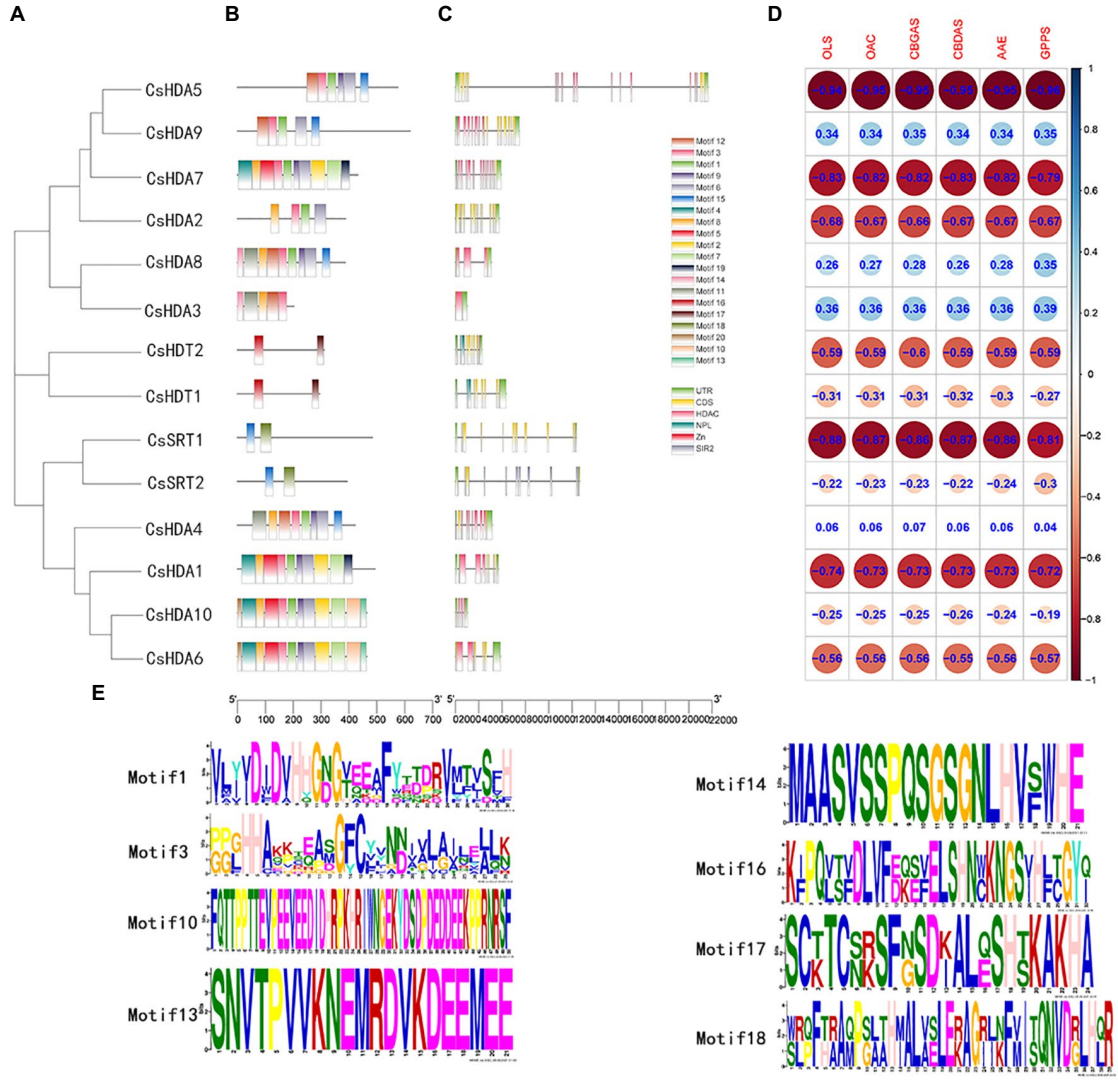
The gene structures and the distribution of conserved motifs of CsHDACs. **(A)** Construction of phylogenetic tree based on the full-length sequence of CsHDAC protein. **(B)** The distribution of the motifs of each CsHDAC protein. **(C)** The gene structure and distribution of the conserved domains of CsHDACs. Green boxes, unrelated area; yellow boxes, exons; black lines, introns; pink boxes, HDAC domains; blue boxes; blue boxes, NPL domains; red box, zinc finger domain; and gray boxes, SIR2 domains. **(D)** Co-expression analysis of CsHDACs and key genes in the cannabinoid synthesis pathway. **(E)** The sequence information for representative motifs.

The conserved motifs of proteins may be related to their transcriptional activity, protein interactions, and nuclear localization (Ikeda and Ohme-Takagi, 2009; Causier et al., 2012). Thus, we studied the conserved motifs of all CsHDAC proteins combined with the evolutionary relationship of hemp (Figure 3A), and 20 conserved motifs were predicted (Figure 3B). The RPD3/HDA1 subfamily included numerous conserved motifs, while the SIR2 and HD2 subfamilies contained relatively fewer conserved motifs. According to the analysis of the conserved motifs, motifs 1 and 3 partly represented the distribution of the HDAC-conserved domain and were shared by all 10 members of the RPD3/HDA1 subfamily. Motifs 10, 13, and 14 were highly conserved and were included only in the Class II subgroup. The conserved motifs 16 and 17 were identified as a characteristic NPL domain, a structure that is unique to the HD2 subfamily. Motif 18 corresponded to the SIR2 conserved domain, which only existed in the SIR2 subfamily (Figure 3E). Multiple sequence comparison of important conserved motifs is shown in Supplementary Figure S2. Other conserved motifs were also found in CsHDACs, while the action mechanism of these motifs has not been analyzed. Overall, the conserved motif composition and gene structure within each family of CsHDAC members had a high similarity, and the results of the phylogenetic analysis advocated the validity of the population classification.

We investigated the exons and introns from their number and distribution to study the structural composition of the *CsHDAC* genes (Figure 3C). Two members of the SIR2 subfamily had eight exons, and two members of the HD2 subfamily contained 10 exons. The coding sequences of the entire RPD3/HDA1 subfamily were interrupted by introns, with the number of exons in the range of 1–17. The *CsHDAC* genes had three main domains, which were specifically distributed into three subfamilies (Figure 3B). The SIR2, RPD3/HDA1, and HD2 subfamilies consisted of the SIR2, HDAC, and NPL domains, respectively. Members of the HD2 subfamily of *C. sativa* had conserved MEFWG pentapeptide at the N-terminus and possibly had a zinc finger at the C-terminus. Generally, the number of exons within each subgroup was similar. The domain of each subfamily was conserved, although the structures of members within a particular subfamily were different. In addition, we analyzed the co-expression analysis of CsHDACs and key genes in the cannabinoid synthesis pathway (Figure 3D; Supplementary Table S1). We found that *CsHDA1*, *CsHDA5*, *CsHDA7*, and *CsSRT1* have obvious correlations with key genes.

### Chromosomal Localization of *CsHDACs*

**Figure 4:**
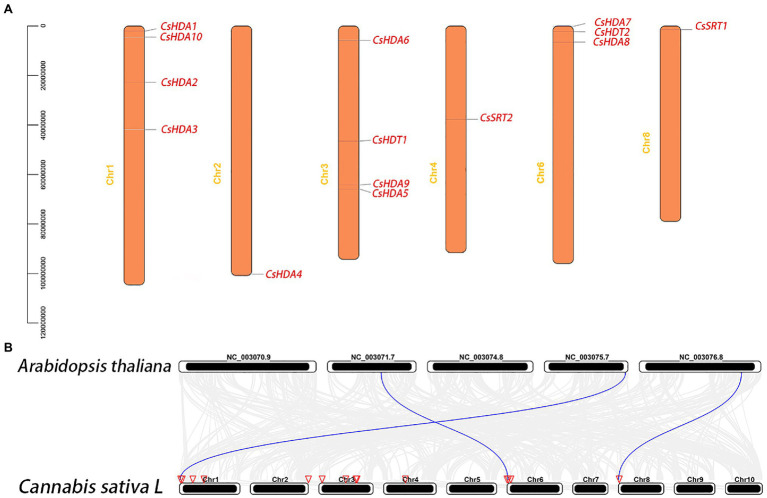
**(A)** Chromosomal locations of the *CsHDAC* genes. The y axis shows the length of the chromosome (unit: bp). **(B)** Synteny analysis of HDAC genes between *Cannabis sativa* and *A. thaliana*. The red triangle side indicates the position of the HDAC gene on the chromosome. The blue lines represent the syntenic HDAC gene pairs.

Chromosome mapping of the *CsHDAC* genes was performed using the latest *C. sativa* genome database. A total of 14 *CsHDAC* genes were unevenly distributed on six chromosomes (Figure 4A). Among them, chromosomes 1, 2, 3, 4, 6, and 8 harbored 5, 1, 4, 1, 3, and 1 genes, respectively. While chromosomes 5, 7, 9, and 10 did not contain any *HDAC* gene. We further studied the homologous genes of CsHDAC within and between species. No homologous genes are found on the chromosomes of *C. sativa*. The results of collinearity analysis between *C. sativa* and *A. thaliana* showed three orthologous genes (Figure 4B).

### *CIS*-Acting Element Analysis of *CsHDACs*

**Figure 5:**
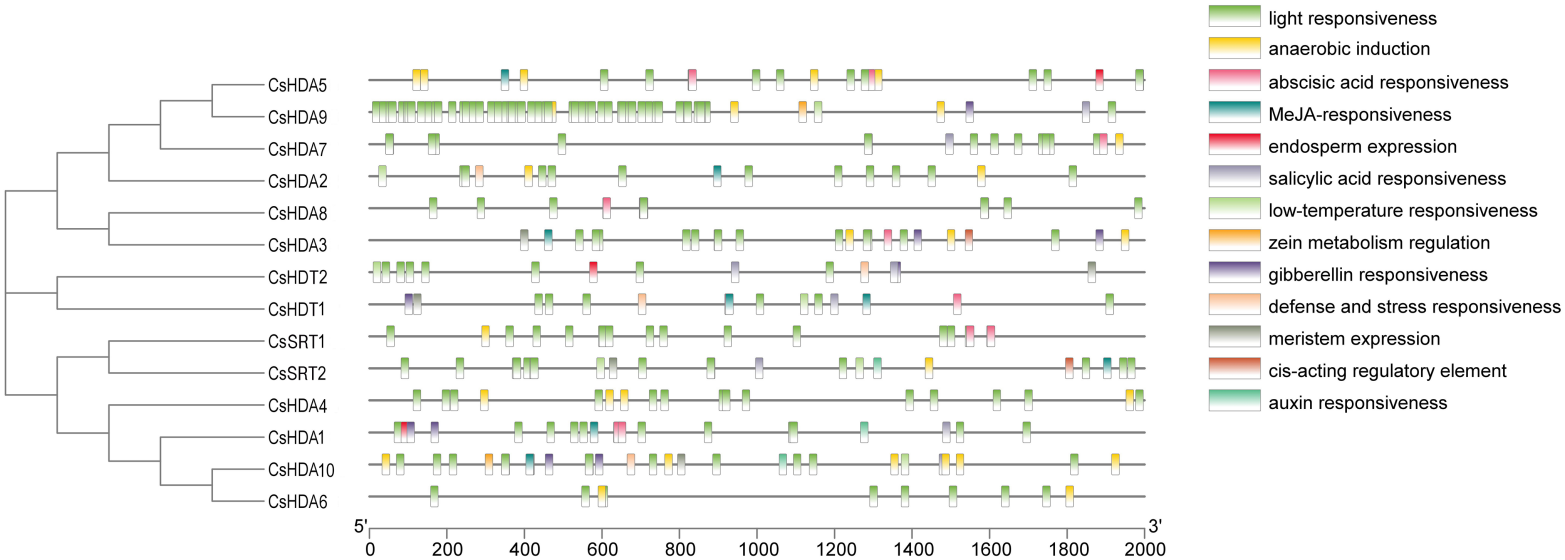
*CIS*-acting elements of the *CsHDAC* genes.

*Cis*-acting element analysis was performed on the CsHDAC gene family members, and the results were visualized (Figure 5). The HDAC gene family contained 13 *cis*-type elements, and each member of the family contained multiple response elements. Among them, *HDAC9* contained many light-responsive elements, suggesting that *HDAC9* may be highly sensitive to light stimuli. *HDA10* contained the largest number of cis-elements, with six *cis-*elements, whereas *HDA8* had the least number of *cis*-elements, with only two *cis-*elements.

### Alternative Splicing Analysis and Gene Expression Patterns

**Figure 6:**
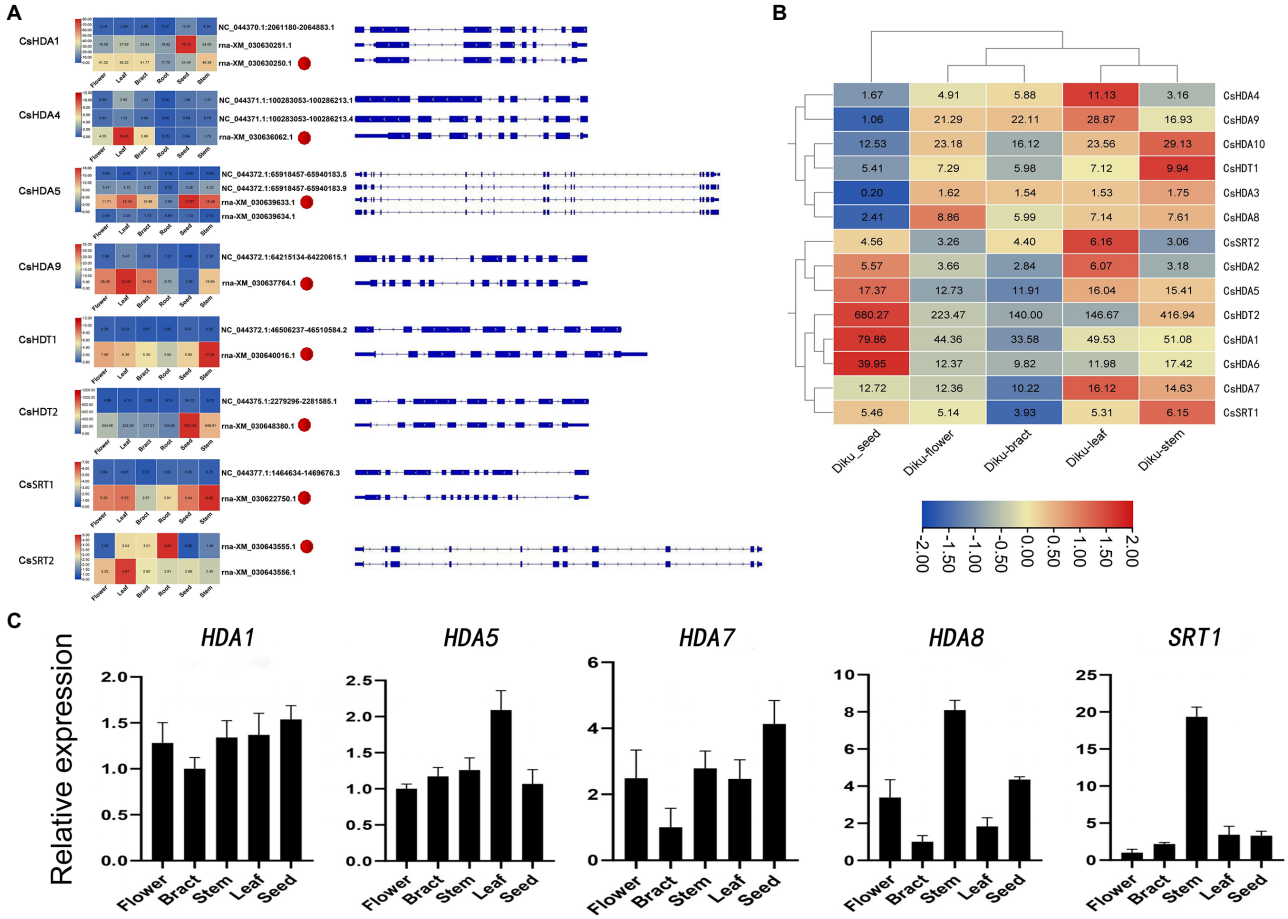
**(A)** Alternative splicing isoforms and the expression pattern of eight *CsHDAC* genes. Red dots refer to the selected reference isoforms. **(B)** The gene expression profile of *CsHDACs* in the seed, flower, bract, leaf, and stem. The row scale on the values has been performed, and the FPKM value of each gene was presented in the figure. **(C)** Expression patterns of the selected *CsHDACs* genes in the flower, bract, stem, leaf, and seed. The characters on the x axis indicate different organs and tissues. The y axis shows the relative level of gene expression. The *EF1-α* gene was used as an internal reference.

Alternative splicing, which causes polymorphisms in the function and structure of proteins, is extensive in eukaryotic organisms (Chaudhary et al., 2019). Therefore, we performed hypomorphic detection and prediction on the basis of data obtained *via* second-generation sequencing and third-generation sequencing and identified 21 splicing events in eight HDAC genes (Figure 6A). Compared with the eight selected reference isoforms, splicing events included seven intron retentions (IR), three alternative 3′ splice site (A3SS; AA), one exon skipping (ES), and two other isoforms (“others”). One gene may generate different alternative splicing events. For instance, NC_044372.1: 65918457–65940183.9 (*CsHDA5*) contained two cutting types: IR and “others.” The expression of different transcripts of the same gene differed. In other words, some transcripts dominated the expression of the gene in all tissues. For instance, rna-XM_030622750.1 (*CsSRT1*) had higher expression in all tissues than the other transcripts. Additionally, different transcripts may be enriched in various tissues. For instance, the rna-XM_030643555.1 (*CsSRT2*) had the highest expression in roots, while another splice variant, rna-XM_030643556.1 (*CsSRT2*), had the highest expression in leaves.

To explore the expression patterns of the *CsHDAC* gene family in different organs, we drew heat maps based on the FPKM calculated from RNA-seq data of seeds, flowers, stems, leaves, and bracts of DK (Figure 6B). *CsHDA1*, *5*, *7,* and *13* were commonly expressed in all tissues. *CsHDA1* and *CsHDT2* were highly expressed in all tissues, especially in seeds. The expression of *CsHDA6* was also relatively higher in seeds. *CsHDA10* was highly expressed in the stem, while *CsHDA4* was enriched in leaves. Genes in flowers and bracts had a similar expression pattern, and the majority was expressed at lower levels than in other tissues. Expression patterns of CsHDAC genes in different tissues can be used as the foundation for identifying functional genes in *C. sativa*. According to previously published research, *MaDHAC1*, *AtHDA6*, and *AtHD2* were associated with plant secondary metabolism (Han et al., 2016). Therefore, we selected candidate homologous *CsHDAC* members by constructing NJ trees and performing motif analysis between these genes and *CsHDACs* (Supplementary Figure S3) and verified them *via* qRT-PCR analysis. *CsHDA1* and *CsHDA5* were evenly enriched in all tissues. *CsHDA7*, *CsHDA8,* and *CsSRT1* were less strongly expressed in the bracts (Figure 6C).

### Gene Expression Pattern and Metabolite Determination After TSA Treatment

**Figure 7:**
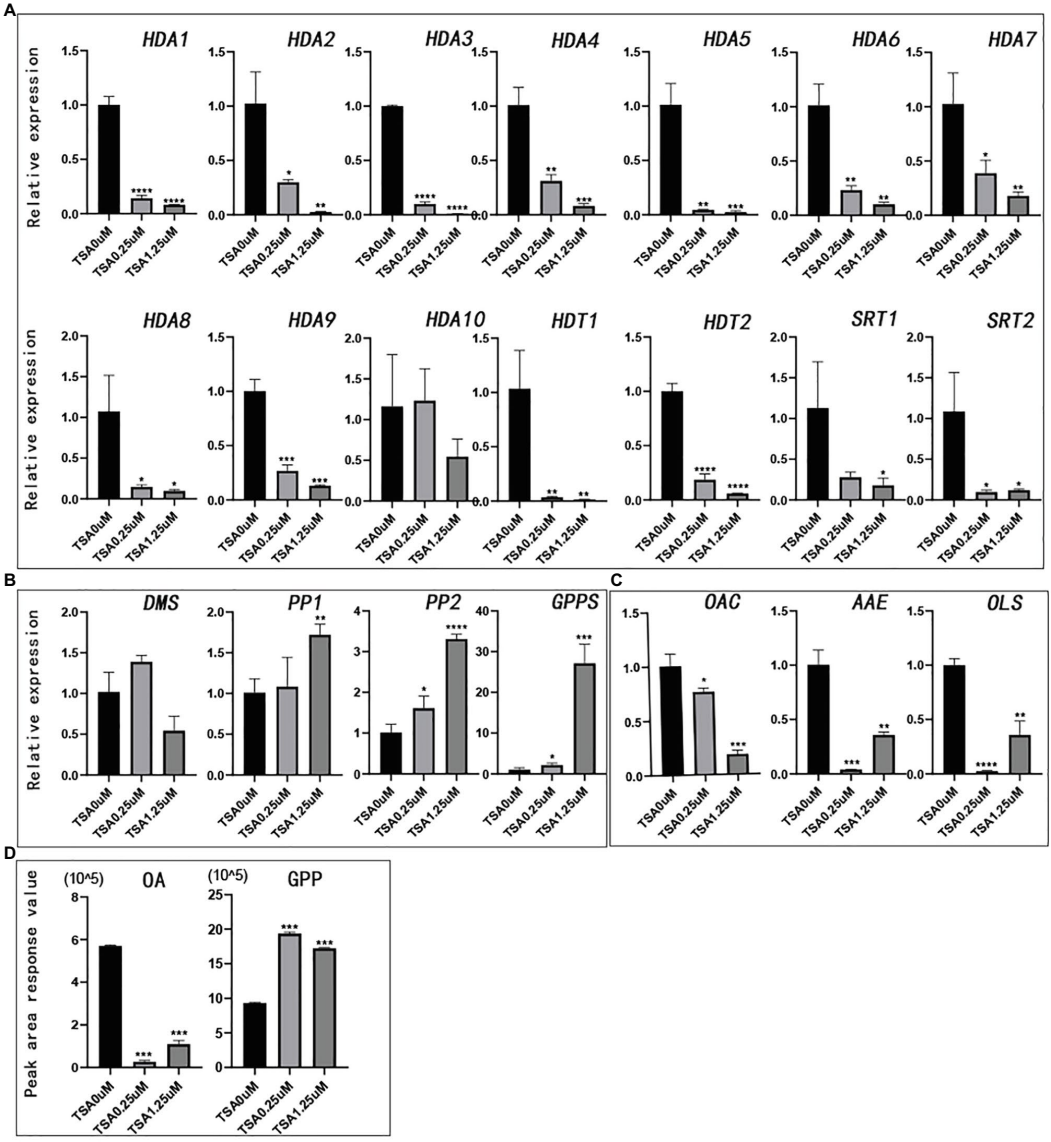
The relative expression of genes and the peak area of synthetic precursors OA and GPP after TSA treatment. The control was performed with 0μm TSA (DMSO matrix) treatment. n=3. **(A)** The relative expression of partial histone deacetylase genes; **(B)** The relative expression of key genes in the MEP pathway; **(C)** The relative expression of key genes in the fatty acid pathway; and **(D)** Peak area of synthetic precursors OA and GPP. ^*^value of *p* <.05, ^**^value of *p* <0.01, ^***^value of *p* <0.001, and ^****^value of *p* <0.0001.

TSA can broadly inhibit the HDAC subfamily (Li et al., 2014; Su et al., 2015). To study whether HDAC is related to the synthesis of cannabinoids, we compared the changes in gene expression in hemp seedlings before and after TSA treatment. HDAC gene expression decreased significantly with increasing concentration of TSA. TSA could effectively inhibit the expression of all the *CsHDAC* genes in the exception of *CsHDA10* (Figure 7A). In this study, two kinds of synthetic precursors were unambiguously identified as OA and GPP, respectively, by comparing the retention times, adductions, and productions with those of authentic standards. We also tested the expression of representative genes in the cannabinoid metabolic pathway and the changes in the synthetic precursors OA and GPP. The expression of genes in the fatty acid synthesis pathway, *OAC*, *OLS*, and *AAE*, was generally downregulated (Figure 7C). However, the expression of *PP1* (HMB-PP reductase1), *PP2* (HMB-PP reductase2), and geranyl diphosphate synthase (*GPPS*) in the MEP pathway increased (Figure 7B). Correspondingly, the content of synthetic precursor OA was reduced, while GPP accumulated (Figure 7D). The above results indicated that TSA inhibited the expression of *CsHDAC* genes and altered the contents of representative genes and synthetic precursors in the cannabinoid synthesis pathway.

## Discussion

Histone deacetylases are widely present in yeast, animals, and plants. The HDAC gene family has been isolated and characterized in several plants, involving 18 genes in *Arabidopsis* (Hollender and Liu, 2008), 18 genes in rice (Fu et al., 2007), 28 genes in soybean (Yang et al., 2018), 11 genes in litchi (Peng et al., 2017), and 15 genes in tomato (Zhao et al., 2015). In this study, we identified 14 *CsHDAC* members and surveyed their gene structures, expression profiles, phylogenetic relationships, and conserved motifs. CsHDACs can be classified into three subfamilies, RPD3/HDA1, HD2, and SIR2. The RPD3/HDA1 was the most highly represented CsHDAC subfamily in Arabidopsis (77%) and soybean (64%), suggesting that the RPD3/HDA1 subfamily may have undergone significant expansion during the process of evolution. The HD2 subfamily will be more likely to have a high affinity with the DNA-binding domain or mediate protein-protein interactions if it contains a C-terminal zinc finger (Wu et al., 2000; Dangl et al., 2001). For example, the interaction between *AtHDT2* and *AtDNMT2* (DNA methyl transfer) affected the expression of cold stress genes (Song, 2010), and *PtHDT1* interacted with *PtMYB t*o regulate lignin biosynthesis (Zhang, 2018). In our study, *CsHDT2* had a C2H2 zinc finger in the C-terminus, suggesting that it may participate in protein-protein interactions. Motif 18 was unique to SIR2. We compared the SIR2 subfamily of Arabidopsis, soybean, and hemp, and found that they all contained motif 18, indicating that motif 18 was an important structural feature, that can be used to identify the SIR2 subfamily, and motif 18 could be the SIR2 subfamily structural basis with similar functions (König et al., 2014).

The expression of genes in tissues can help us infer the function of genes. In our study, we predicted and verified the expression of members of the CsHDAC family in different tissues. It was found that the expression of *CsHDA7* in flowers and bracts was relatively low when compared its expression in other organs, which was consistent with the contention that HDACs usually bind inhibitory regulators to exert a negative regulatory effect. *CsHDA1* was evenly distributed in all tissues, and in previous studies, its homologous gene *AtHDA6* significantly contributed to a variety of aspects of plant development and growth, such as abiotic stress responses, jasmine and ethylene signal transmission, and leaf and flower development (Probst et al., 2004; Yu et al., 2011). Both genes were predicted to be localized in the nucleus, suggesting that they were involved in transcriptional regulation. Therefore, *CsHDA1* may be a vital regulator throughout the life course of hemp.

Histone deacetylase is involved in the development of plants, the response to environmental changes, and the synthesis of secondary metabolites (Waterborg, 2011). HDAC usually forms a complex with transcription repressors to regulate target gene expression. In previous research, *AtHDA15* interacted with a negative regulator, *AtHY5*, to repress photomorphogenesis (Jing et al., 2020). *OsHDA1* physically interacted with *OsIDS1* and the transcriptional corepressor *OsTPR1* (topless-related 1) contributing to the repression of *OsLEA1* and *OsSOS1* expression (Cheng et al., 2018). *MaHDAC1* and the transcription repressor *MaERF11* inhibited the expression of genes related to maturation (Han et al., 2016). Combined with previous studies on the regulation of secondary metabolites by HDACs, tissue-specific expression, and co-expression analysis, we initially screened five candidate genes that may be related to cannabinoid regulation. Furthermore, we compared the domain amino acid sequences of CsHDACs and MaHDA1, constructed the NJ tree, and performed BLAST sequence alignment. The results showed that *CsHDA7* and *MaHDA1* had 86.98% amino acid similarity (Supplementary Figure S5), suggesting that *CsHDA7* and *MaHDA1* have a similar effect.

TSA has been shown to inhibit the activity of histone deacetylase. Similarly, the expression of CsHDACs was significantly depressed by TSA during this study. The synthesis of cannabinoids originated from the MEP pathway and fatty acid pathway. Both pathways differed after TSA treatment regardless of gene expression or metabolite accumulation, indicating that TSA can affect the biosynthesis of cannabinoids in several ways. However, it is still unclear whether TSA plays a role *via* inhibiting the expression of related genes or by mediating the structure of chromatin by repressing the activity of histone deacetylase in the cannabinoid synthesis pathway (Supplementary Figure S4).

## Conclusion

We identified 14 CsHDAC family members and analyzed their structural characteristics, evolutionary relationships, and spatial expression patterns. By combining RNA-seq with qRT-PCR analysis, we predicted that five HDACs were associated with cannabinoid synthesis. Furthermore, the reduction of HDAC through TSA treatment directly contributed to the expression of related genes in cannabinoid synthesis and the changes in synthetic precursors of cannabinoids. However, both genes and the synthetic precursors involved in the fatty acid and MEP pathways behaved opposite with the TSA treatment, implying the complexity of HDACs in cannabinoid synthesis. Therefore, the function of candidate genes in cannabinoid synthesis should be thoroughly studied in the future.

## Data Availability Statement

The datasets presented in this study can be found in online repositories. The names of the repository/repositories and accession number(s) can be found in the article/**Supplementary Material**.

## Author Contributions

ML, WS, and YM contributed to conception of the study. LY wrote the manuscript. LY, XM, XY, SC, and JCL conducted the bioinformatics analysis. LY, YZ, and SW contributed to data visualization. LY and YM performed the qRT-PCR experiment. JL, LY, and GQ performed the metabolic experiment. WC completed the data extraction. ML and WS revised the manuscript. SC, WS, and XL contributed to supervision. All authors have read and agreed to the published version of the manuscript.

## Funding

This research received funding from the Scientific and Technological Innovation Project of China Academy of Chinese Medical Sciences (CI2021A04806 and CI2021A04008).

## Conflict of Interest

The authors declare that the research was conducted in the absence of any commercial or financial relationships that could be construed as a potential conflict of interest.

## Publisher’s Note

All claims expressed in this article are solely those of the authors and do not necessarily represent those of their affiliated organizations, or those of the publisher, the editors and the reviewers. Any product that may be evaluated in this article, or claim that may be made by its manufacturer, is not guaranteed or endorsed by the publisher.

## Footnotes

^1^ https://www.ncbi.nlm.nih.gov

^2^ http://planttfdb.cbi.pku.edu.cn/

^3^ https://www.ncbi.nlm.nih.gov/structure/cdd/wrpsb.cgi

^4^ http://web.expasy.org/protparam/

^5^ http://meme-suite.org/tools/meme

^6^ https://www.ncbi.nlm.nih.gov

^7^ http://www.genscript.com/wolf PSORT. HTML

^8^ http://www.plantcare.co.uk/

^9^ https://www.ncbi.nlm.nih.gov

^10^ http://software.broadinstitute.org/software/igv/IGV
