# Supplementary material for: Identification of the Histone Deacetylases Gene Family in Hemp Reveals Genes Regulating Cannabinoids Synthesis
Source: Front Plant Sci. 2021 Oct 20;12:755494. doi: 10.3389/fpls.2021.755494 (PMC8636033; doi:10.3389/fpls.2021.755494)
Supplement: Supplementary file 1 [file Data_Sheet_1.PDF]

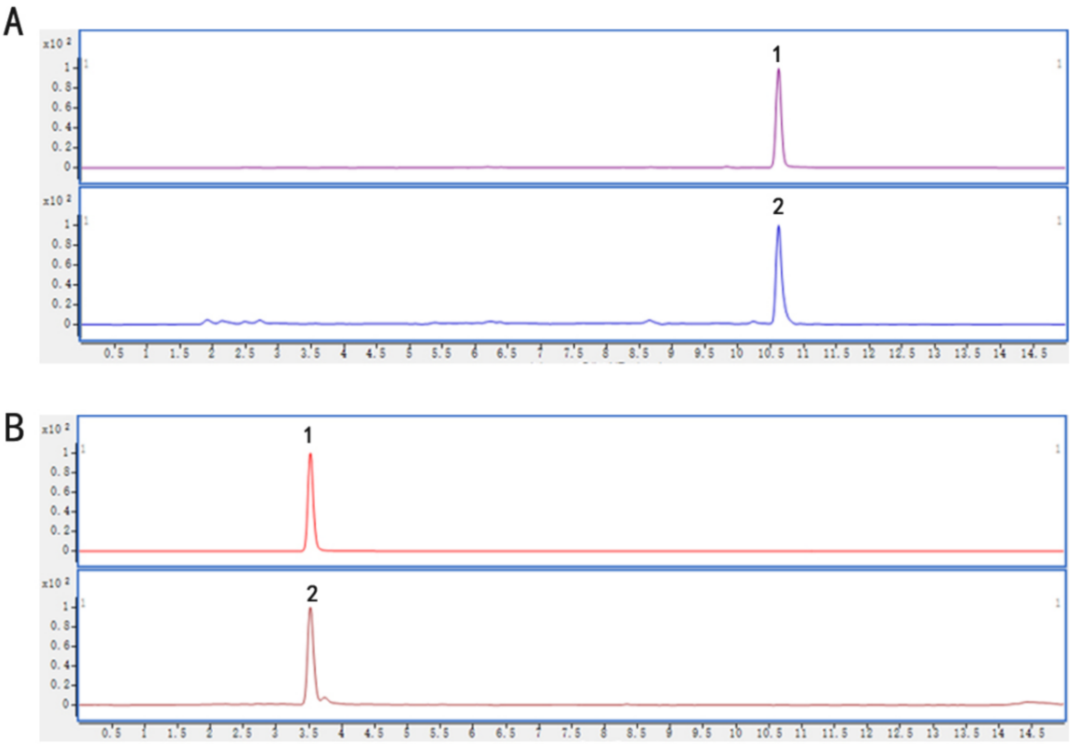

Supplemental Figure l. MRM diagrams of standards and samples. (A) MRM diagram of the OA. 1 represents standard product; 2 indicates sample. (B) MRM diagram of the GPP. 1 shows standard, 2 is sample.The x axis represents the peak time; they axis shows [illegible]

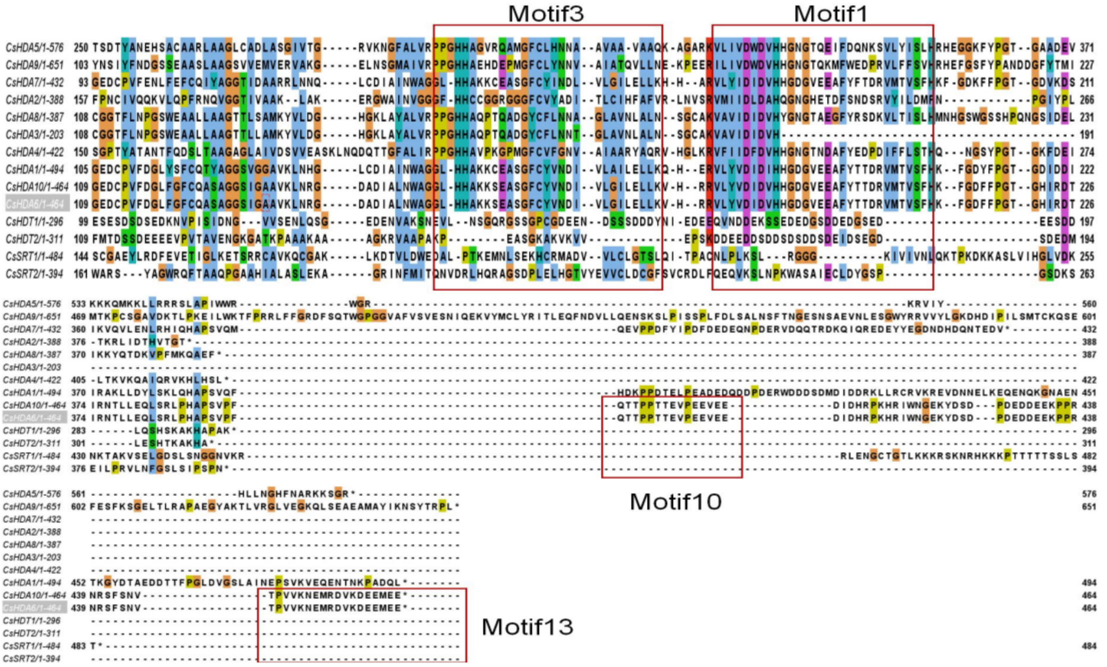

Supplemental Figure 2. Multiple sequence comparison of fourteen HDAC genes. Important motifs are highlighted with red boxes.

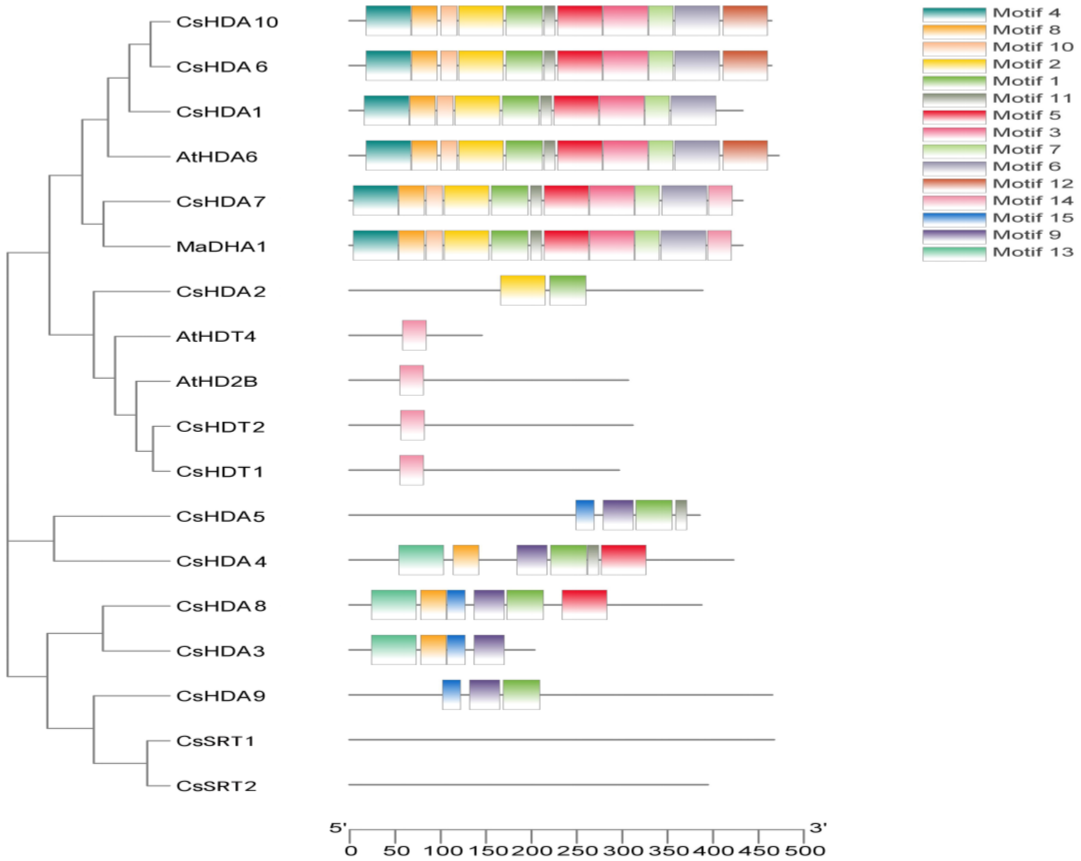

Supplemental Figure 3. Comparison of NJ tree and motif between CsHDACs, AtHDACs and MaHDA1.

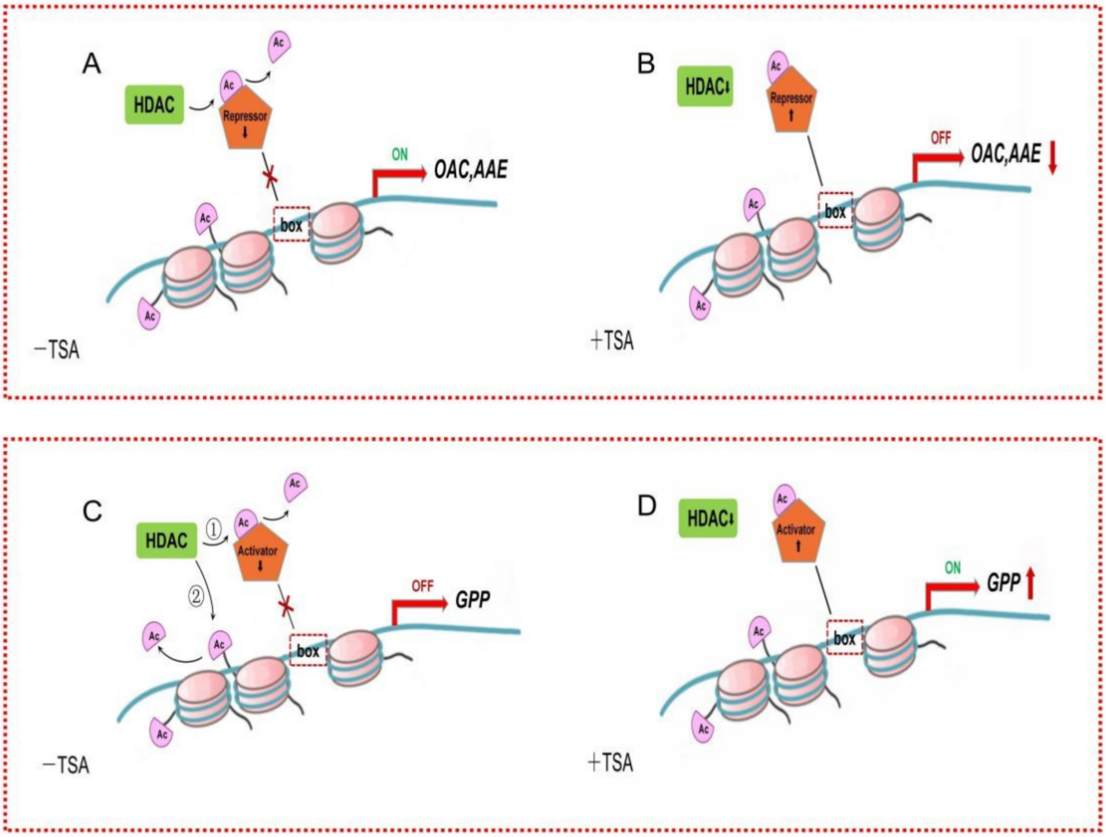

Supplemental Figure 4.A predicted model depicting how TSA regulates cannabinoid-related gene expression via repressing HDAC. In the absence of TSA, repressors of genes involved in fatty acid pathway lose their function via deacetylation (A), while in the presence of TSA, these repressors restore their repression due to the decrease of HDAC, leading to the reduction of cannabinoid related gene expression (B). During MEP pathway, HDACs exert their effect through deacetylating either activators(C①) or promoters (C②), while TSA interrupts their deacetylation (D).

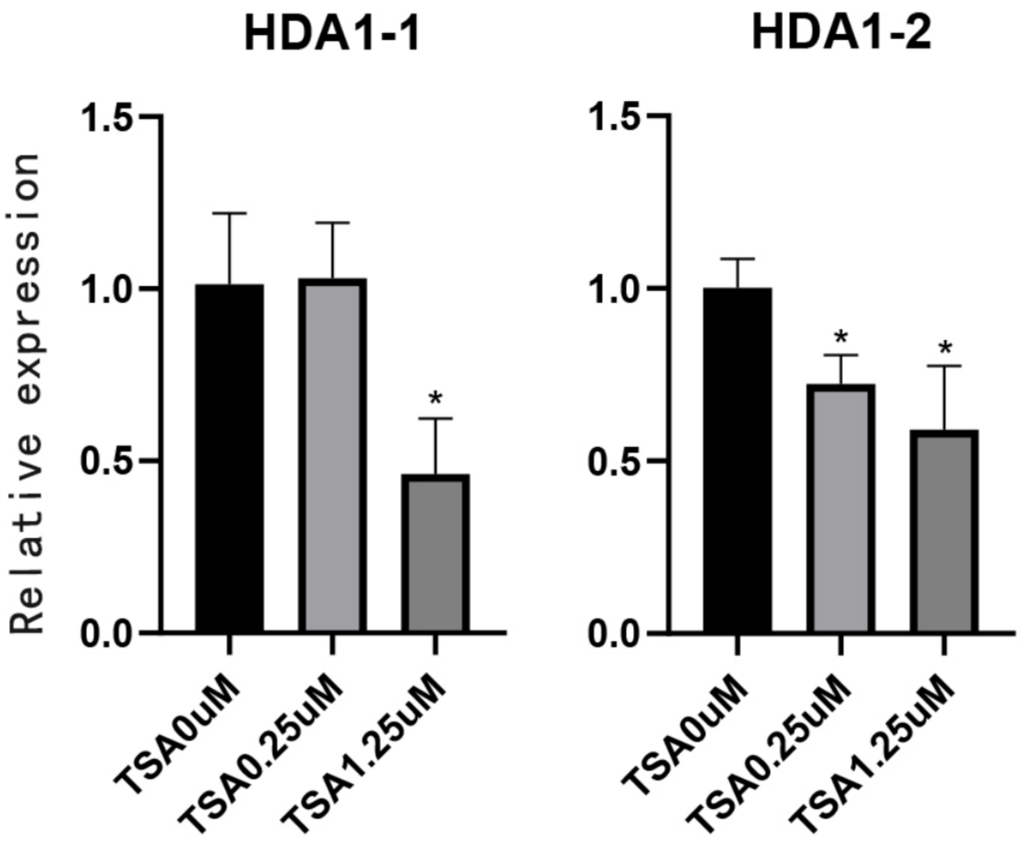

Supplemental Figure 5. The relative expression of HDA1-1 and HDA1-2 after TSA treatment. HDA1-1 refers to the reference isoforms of HDA1, the transcript number is rna-XM_030630250.1; HDA1-2 stands for the "ES" isoforms of HDA1, and the transcript number is rna-XM 030630251.1.

Name	OLS	OAC	CBGAS	CBDAS	AAE	GPPS
CsHDA1	0.15	0.16	0.16	0.16	0.16	0.17
CsHDA2	0.21	0.22	0.23	0.22	0.21	0.22
CsHDA3	0.55	0.55	0.55	0.56	0.55	0.52
CsHDA4	0.92	0.93	0.92	0.92	0.93	0.94
CsHDA5	0.02	0.01	0.01	0.02	0.01	0.01
CsHDA6	0.33	0.33	0.33	0.33	0.33	0.32
CsHDA7	0.09	0.09	0.09	0.09	0.09	0.11
CsHDA8	0.68	0.63	0.64	0.67	0.64	0.56
CsHDA9	0.57	0.57	0.57	0.58	0.57	0.56
CsHDA10	0.68	0.70	0.69	0.68	0.70	0.76
CsHDT1	0.61	0.62	0.61	0.60	0.62	0.66
CsHDT2	0.29	0.29	0.29	0.29	0.29	0.29
CsSRT1	0.05	0.06	0.06	0.05	0.06	0.10
CsSRT2	0.73	0.69	0.71	0.72	0.70	0.63

Supplemental Table 1. P value for co-expression analysis of HDACs and key gene
